# Electronic nicotine delivery systems (ENDS) use across the menstrual cycle and oral contraceptive regimen: A proof-of-concept intensive longitudinal study

**DOI:** 10.1016/j.dadr.2025.100350

**Published:** 2025-06-13

**Authors:** Chrystal Vergara-Lopez, George D. Papandonatos, Margaret H. Bublitz, Alicia M. Allen, Laura R. Stroud

**Affiliations:** a Department of Psychiatry and Human Behavior, The Warren Alpert Medical School of Brown University, Providence, RI, USA; b Center for Behavioral and Preventive Medicine, The Miriam Hospital, Brown University Health, Providence, RI, USA; c Department of Biostatistics, School of Public Health, Brown University, Providence, RI, USA; d Department of Medicine, The Warren Alpert Medical School of Brown University, Providence, RI, USA; e Department of Obstetrics and Gynecology, The Warren Alpert Medical School of Brown University, Providence, RI, USA; f Department of Family and Community Medicine, College of Medicine-Tucson, University of Arizona, Tucson, AZ, USA; g Department of Epidemiology and Biostatistics, Mel and Enid Zuckerman College of Public Health, University of Arizona, Tucson, AZ, USA

**Keywords:** Electronic nicotine delivery systems, Nicotine use, Ovarian hormones, Menstrual, Cycle, Oral contraceptives, Estradiol, Progesterone

## Abstract

**Introduction:**

Exogenous and endogenous ovarian hormones (e.g., estradiol, progesterone) may influence nicotine use. Prior research has focused on combustible cigarettes and yielded mixed results, which may be due to a lack of granular assessment of nicotine use across the menstrual cycle or oral contraceptive (OC) regimen. We conducted a small proof-of-concept study on Electronic Nicotine Delivery Systems (ENDS). Our goals were to examine the utility of intensive longitudinal methods to assess ENDS use in a ~month long protocol, and explore ENDS use levels and variability among naturally cycling (NC) individuals and those using OCs.

**Methods:**

There were 12 NC participants (*M*_*age*_=22) and 7 participants using OCs (M_age_=21). ENDS occasions were assessed 4 times a day across the protocol.

**Results:**

On average, the NC group completed 77 % and the OC group completed 86 % of ENDS assessments. The average number of missing data was 2.2 days (*SD*=2.9). Time-Varying Effect Modeling (TVEM) examine changes in links between variables over time. TVEM revealed increases in ENDS use coinciding with rises in estradiol across the menstrual cycle. In contrast, ENDS use was consistent in the OC group.

**Conclusions:**

Preliminary evidence indicates that ENDS use among NC individuals varies as a function of natural fluctuations in ovarian hormones while OCs appear to lower and stabilize ENDS use. Despite the small sample, this study suggests that intensive longitudinal methods are useful for examining links between the menstrual cycle, OCs, and ENDS use. This proof-of-concept research may galvanize mechanistic and intervention research on ovarian hormones and ENDS use.

## Highlights

• ENDS use may vary as a function of naturally occurring fluctuations in estradiol across the menstrual cycle.
• Tentative evidence suggests that oral contraceptives may lower and stabilize ENDS use.
• Intensive longitudinal methods are useful for examining links between the menstrual cycle/OC regimens and ENDS use.

## 1 Introduction

Exposure to ovarian hormones, either endogenously via the menstrual cycle or exogenously via oral contraceptives (OC), are implicated as critical sex-specific factors impacting nicotine use (Weinberger et al., 2015, Allen et al., 2019, Wetherill et al., 2016). Yet there is a paucity of research focused on Electronic Nicotine Delivery Systems (ENDS; non-combustible devices that heat a nicotine-containing freebase liquid or salt to form an inhalable aerosol) (Breland et al., 2017). Over the last decade, ENDS have become the most common form of nicotine use by young adults (ages 18–24) in the United States, far surpassing traditional combustible cigarettes (Burt and Li, 2020). Thus, expanding research to test the link between ovarian hormones and ENDS use is urgently needed.

The menstrual cycle is characterized by the rise and fall of endogenous estradiol and progesterone (Schiller et al., 2016). The onset of menses marks the first day of the cycle (and the beginning of the follicular phase), and approximately two weeks prior to the end of the cycle ovulation (i.e., release of an ovum from an ovary) marks the onset of the luteal phase (Allen et al., 2016). The follicular and luteal phases have subphases. The early follicular subphase has low estradiol and progesterone, while the late follicular subphase displays a rise in estradiol and low progesterone. The early luteal subphase is marked by rising progesterone and initial low levels of estradiol with a second but attenuated peak. In the late luteal subphase, both progesterone and estradiol decrease (which can be accompanied by negative affect for some people) (Eisenlohr-Moul, 2019). A typical cycle ranges between 22 and 35 days with both intra- and inter-individual variability (Fehring et al., 2006).

Combined-type monophasic OCs containing synthetic forms of estrogen and progesterone components are the most used hormonal contraceptives (National Center for Health Statistics, 2023, Verma et al., 2021). Commonly there are three active pill weeks (days 1–21) that contain a consistent dose of ethinyl estradiol and progestin. The fourth week is a placebo week and does not contain hormones. There are many brands and formulations; however, all contain ethinyl estradiol (a synthetic estradiol) and vary on the specific form of synthetic progesterone. These contraceptives stabilize the production of endogenous estradiol and progesterone by blocking ovulation and leading to a low and stable estradiol to progesterone ratio (Hampson, 2020).

Most of the research linking ovarian hormones to nicotine use has focused on combustible cigarettes, yielding mixed results. Some studies suggest the follicular phase increases susceptibility to nicotine use and is linked to poorer smoking cessation, while other studies implicate the luteal phase as conferring more risk for nicotine use and/or poor cessation (Weinberger et al., 2015, Wetherill et al., 2016). There have been very few studies focused on OCs. One study showed that OCs may aid cessation (Allen et al., 2018); while others link OCs to increased nicotine metabolism and potentially use (Allen et al., 2019). Many past studies have relied on between-subject designs, retrospective self-report of menses or OC use, and a dichotomized menstrual cycle, which provides a snapshot of the impact of the menstrual cycle or OC regimen on nicotine use. Mixed findings may be due to a lack of granular assessment of nicotine use across the menstrual cycle or OC regimen. Indeed, current gold-standard methods for assessing ovarian hormones require challenging-to-employ prospective, repeated-measures methods (Schmalenberger et al., 2021). To address the current literature gap, we conducted a proof-of-concept study to (1) examine the utility of intensive longitudinal methods to assess ENDS use across the menstrual cycle or OC regimen, and (2) explore ENDS use levels and variability among naturally cycling (NC) individuals and those using combined-type monophasic OCs.

## 2 Methods

### 2.1 Participants

Participants were drawn from a centralized recruitment effort for studies on substance use among young adults. Social media campaigns as well as advertisements in local establishments were used to recruitment participants in Rhode Island, Massachusetts, or Connecticut (United States). We recruited participants from a screened pool that consisted of individuals that were 18–25 years-old and who self-reported ENDS use “every day” or “at least two days per week” (of which 45 % reported menstrual cycles between 22 and 35 days, and 41 % used hormonal birth control). Based on self-report individuals were categorized into the NC group if they experienced menses every 22–35 days and were not using any hormonal contraceptives (cycle length was prospectively confirmed). Among those in the screened pool using hormonal birth control, 75 % self-reported using combined monophasic oral contraceptives consisting of 21 days of active synthetic hormones followed by 7 days of placebo. Thus, we recruited from this group of the most common OC formulation (Verma et al., 2021) (there was no endorsement for the progestogen-only pills). There were 12 NC participants and 7 participants using OCs containing ethinyl estradiol and a synthetic progestin.

### 2.2 Study procedures

We obtained the first day of the last menstrual period or the first day of the last start of an OC pill packet prior to enrollment via retrospective self-report. If individuals were unsure of these dates or if recall led to incorrect projections, staff conducted prospective recruitment outreach to identify these dates for the next cycle or OC regimen. This information was used to schedule the one-time baseline enrollment session. NC participants enrolled during menses (*M*_*cycleday*_=2.9, *SD*_*cycleday*_=1.2). OC participants enrolled during the inactive/placebo week of a pill packet. These procedures aimed to capture Day 1 of the menstrual cycle and Day 1 of an OC regimen in order to follow participants for an entire natural cycle or OC regimen. At the one-time in person session participants completed informed consent, baseline measures, and training for an intensive longitudinal protocol. Participants were then followed across their menstrual cycle (ranging from 24 to 34 days in this sample) or one OC regimen (1st active pill of the next packet marked day 1 and they were followed for 28 days). This intensive longitudinal protocol was delivered via a smartphone “app” on the LifeData platform (https://www.lifedatacorp.com) which allowed for live compliance tracking. Staff reached out with reminders if a participant missed more than two assessments. The protocol (# 1243650) was approved by a local institutional review board.

### 2.3 Study measures

Baseline measures including demographic information (e.g., age, race/ethnicity, gender, sexual orientation), reproductive health history, characteristics related to ENDS, other nicotine products, as well as alcohol and cannabis were assessed using items adapted from the Population Assessment of Tobacco and Health (PATH) study (United States Department of Health and Human Services). Participants reported their typical wake schedule, which was used to schedule four fixed-time app assessments covering waking hours. During the first assessment participants reported the number of ENDS occasions since waking up. The other three assessments required participants to report the number of ENDS occasions since the last assessment. Participants received one-on-one instruction on the study's definition of an ENDS occasion and how to use the app. They were also provided with a training manual. ENDS occasions were defined as the participants' perceived beginning and end of a series of at least two puffs (Reed et al., 2023, Addicott et al., 2023, Berg et al., 2019, Cooper et al., 2019). Participants where coached to notice that occasions were separated by engaging in another activity after using the ENDS. The optimal number of app observations varied by participant due to differences in cycle length. We collected a range of 84–110 observations per participant. Additionally, each day we assessed vaginal bleeding (Schmalenberger et al., 2021). To determine menstrual cycle day, we followed gold-standard recommendations by utilizing assessment of the previous cycle’s start of menses and the current cycle’s start of menses and then employed forward and backward count strategies (Schmalenberger et al., 2021, Allen et al., 2016). For the forward count method, we obtained retrospective report of the 1st day of the last menses to predict the 1st day of the next menses which was prospectively verified by participant self-report. Participants were then followed daily via the app during this cycle, providing information on the next 1st day of menses. Menstrual cycle day was established by counting the number of days from the onset of menses to the next menses (the beginning of a third cycle). A backward count was also employed by retrospectively counting backwards from the onset of menses of the third cycle to determine the menstrual cycle day of the second cycle. Thus, we had information for the onset and offset of menses for three cycles and participants were followed via the app during the second of these cycles. We also had prospective data on participants that allowed us to determine the first day they started a new OC pill packet.

### 2.4 Data analytic plan

We descriptively summarize demographic information and other characteristics.

Next, on an exploratory basis, we fitted time-varying effect models (TVEM), (Tan et al., 2012) to intensive longitudinal data utilizing the TVEM R package (Dziak et al., 2023). TVEM uses penalized B-splines to estimate time-varying associations between pairs of variables in a flexible manner, without making assumptions on the nature of these associations. In these analyses, the dependent variable was the number of ENDS use occasions per day, whereas the independent variable was time itself. We employed two different time scales for the NC and OC groups, with the time origin (day 1) set to the beginning of the menstrual cycle (i.e., the first day of menses) or the first day of use of a new OC pill packet, respectively.

## 3 Results

### 3.1 Study sample descriptive findings

Table 1 provides sample characteristics by group. The NC group was comparable in age (*M*=22 years; *SD*=2.0) to the OC group (*M*=21 years; *SD*=2.0). The majority of the sample (68 %) was Non-Hispanic White; 100 % identified as women; 63 % identified as heterosexual, and 26 % identified as bisexual. The average age of first menses was 12 years (*SD*=1.0). One participant had a past pregnancy, and no participants had children. Among the OC group the average age for initiating use of hormonal contraceptives was 16 years old (*SD*=1.0) and all participants had utilized the same oral contraceptive regime for at least 1 year. Most participants used ENDS every day (NC=75 %, OC=71 %), and 25 % of NC individuals, and 29 % of participants on OCs utilized combustible cigarettes in the past 6 months. Furthermore, most participants had used alcohol (NC=92 %, OC=100 %) and cannabis (NC=92 %, OC=71 %) in the past 6-months. Due to the limited sample size, caution is warranted against confident between-group inferences. However, descriptively, ENDS occasions per day across a menstrual cycle (including ovulation and menses) were nearly 2X higher in the NC group (*M*=15.8, *SD*=15.1) than among those utilizing oral contraceptives (*M*=8.8, *SD*=7.2). Next, we report ENDS occasions by menstrual cycle subphases (early follicular, *M*=20.0, *SD*=20.9; mid/late follicular, *M*=23.0, *SD*=22.4; early luteal, *M*=19.6, *SD*=20.5; mid/late luteal, *M*=20.0, *SD*=21.7). Lastly, in terms of protocol compliance, on average the NC group provided 77 % and the OC group provided 86 % of possible observations, and across the groups the average number of missing data was 2.2 days (*SD*=2.9).

**Table 1:** Sample characteristics by group.

**Demographic Characteristics**		
**Characteristic**	**Naturally Cycling Participants**	**Oral Contraceptive Participants**
Age	*Mean*= 22 (*SD*=2.0)	*Mean*= 21 (*SD*=2.0)
Gender		
Woman	100 %	100 %
Man	0 %	0 %
Non-binary	0 %	0 %
Prefer to self-describe	0 %	0 %
Ethno-Racial Identity		
Non-Hispanic White	58 %	86 %
Black	0 %	14 %
Latina/e	17 %	0 %
Multi-Racial	25 %	0 %
Sexual Orientation		
Heterosexual	50 %	86 %
Bisexual	42 %	0 %
Lesbian/Gay	8 %	0 %
Pansexual	0 %	14 %
Financially Independent	50 %	14 %
Non-Financially Independent	50 %	86 %
Household Yearly Income		
$0- $10,000	16.67 %	14 %
$10,000- $20,000	8.33 %	29 %
$20,000- $30,000	16.67 %	14 %
$30,000- $40,000	16.67 %	0 %
$40,000- $50,000	8.33 %	0 %
$50,000- $60,000	8.33 %	0 %
$60,000- $70,000	0 %	0 %
$70,000- $80,000	0 %	0 %
$80,000- $90,000	8.33 %	0 %
> $90,000	8.33 %	14 %
Prefer Not to Answer	8.33 %	29 %
Family of Origin's Yearly Income		
$0- $10,000	0 %	0 %
$10,000- $20,000	0 %	14 %
$20,000- $30,000	8.33 %	0 %
$30,000- $40,000	0 %	0 %
$40,000- $50,000	25 %	14 %
$50,000- $60,000	8.33 %	14 %
$60,000- $70,000	0 %	14 %
$70,000- $80,000	8.33 %	29 %
$80,000- $90,000	8.33 %	0 %
> $90,000	33.33 %	0 %
Prefer Not to Answer	8.33 %	14 %
**Electronic Nicotine Delivery Systems (ENDS) Use Characteristics**		
Age of Initiation		
13–14 Years Old	8 %	0 %
15–16 Years Old	25 %	43 %
17 Years or Older	67 %	57 %
Frequency of Use: Past 6-Months		
Every day	75 %	71 %
A few days a week	25 %	29 %
Quantity of Use: Past 6-Months		
1 time per day	0 %	0 %
2 times per day	0 %	0 %
3–4 times per day	8.33 %	14 %
5–6 times per day	8.33 %	15 %
7–8 times per day	8.33 %	0 %
9–10 times per day	8.33 %	0 %
11 + times per day	67 %	71 %
Brand		
Juul	50 %	43 %
Puff Bar	33 %	14 %
Luto	8 %	0 %
ESCO Ba	8 %	14 %
Hyde Bar	8 %	0 %
Gippro	0 %	14 %
Don’t Know	8 %	29 %
Type		
Pod	50 %	43 %
Disposable	58 %	29 %
Cartridge	0 %	14 %
Don’t Know	8 %	14 %
Typical Concentration of Nicotine		
50 + mg or 5.0 %+	67 %	86 %
Don’t Know	33 %	14 %
ENDS Dependence	*Mean*= 2.04 (*SD*=.83)	*Mean*= 1.57 (*SD*=1.07)
**Combustible Cigarette Use Characteristics**		
Ever Used	58 %	29 %
Used in the Past 6-Months	25 %	29 %
Frequency of Use: Past 6-Months		
Once a month or less	8 %	14 %
2–3 days a month	8 %	0 %
Every day	8 %	14 %
Quantity of Use: Past 6-Months	*Range*= 2–3	Range= 0.5–6
Typical # Cigarettes on use days		
**Other Nicotine Product Use Characteristics**		
Ever Used: Cigar	17 %	57 %
Past 6-Months: Cigar	0 %	0 %
Ever Used: Little Cigar	25 %	0 %
Past 6-Months: Little Cigar	0 %	0 %
Ever Used: Cigarillo	17 %	14 %
Past 6-Months: Cigarillo	0 %	0 %
Ever Used: Chewing Tobacco	8 %	43 %
Past 6-Months: Chewing Tobacco	8 %	43 %
Ever Used: Hookah	50 %	0 %
Past 6-Months: Hookah	25 %	0 %
Ever Used: Other	0 %	0 %
Past 6-Months: Other	0 %	0 %
**Cannabis Use Characteristics**		
Ever Used	100 %	100 %
Used in the Past 6-Months	92 %	71 %
Frequency of Use: Past 6-Months		
Monthly or less	9 %	40 %
2–4 times a month	18 %	0 %
2–3 times a week	0 %	0 %
4 or more times a week	73 %	60 %
Quantity of Use: Past 6-Months		
Typical # of hours “stoned” or “high” on use days		
Less than 1	0 %	0 %
1 or 2	64 %	40 %
3 or 4	27 %	40 %
5 or 6	9 %	0 %
7 or more	0 %	20 %
**Alcohol Use Characteristics**		
Ever Used	100 %	100 %
Used in the Past 6-Months	92 %	100 %
Frequency of Use: Past 6-Months		
Monthly or less	0 %	43 %
2–4 times a month	45 %	14 %
2–3 times a week	45 %	43 %
4 or more times a week	9 %	0 %
Quantity of Use: Past 6-Months		
Typical # of drinks on use days		
1 or 2	27 %	57 %
3 or 4	36 %	14 %
5 or 6	36 %	29 %
7–9	0 %	0 %
10 or more	0 %	0 %

*Note*: The “prefer not to answer” option was provided for all assessments but not depicted when there was 0 endorsement for presentation clarity. Financial Independence is defined as the participants’ main source of income is from a personal or spouse/partner job. Non-financial independence is defined as at least 50 % of income comes from parent(s) or family of origin. Household yearly income is inclusive of participants’ income and spouse or partner income. For age of initiation younger ages were assessed but not endorsed, thus, not depicted. Frequency and quantity of use reflects retrospective report of patterns of use in the past 6-months assessed at baseline. Participants were asked to report on the usual concentration of nicotine in the electronic nicotine delivery system (ENDS) product. To assess this question options lower than 50 +  mg or 5.0 %+  were provided but not endorsed, thus, not depicted. Participants could endorse more than one brand or type of ENDS. ENDS dependence was assessed by the 4-item version of the E-cigarette Dependence Scale with possible range of scores from 0 to 4 (Medvescek and Allen, 2023).

### 3.2 Time-varying effect models

**Fig. 1:**
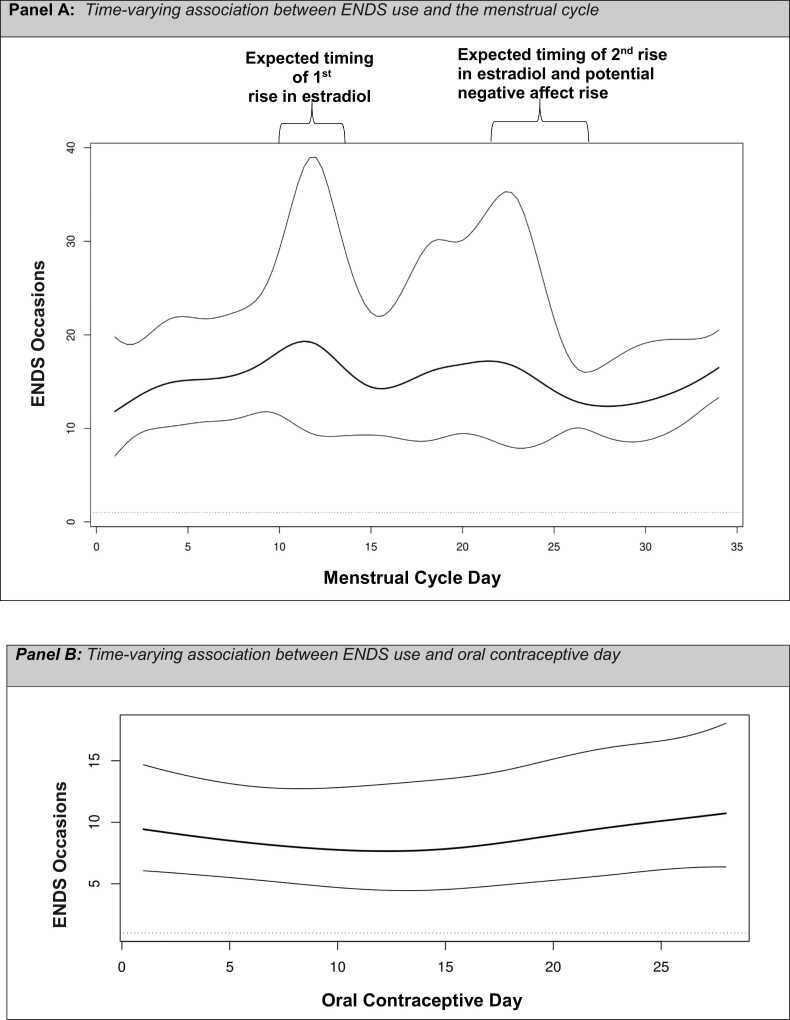
Panel a demonstrates the main effect of time, in this case menstrual cycle day, on electronic nicotine delivery systems (ENDS) use among naturally cycling participants accompanied by pointwise 95 % confidence intervals. Panel b demonstrates the main effect of time, in this case day of an oral contraceptive regimen, on ENDS use among participants utilizing oral contraceptives accompanied by pointwise 95 % confidence intervals.

Main effects of time on ENDS use in both the NC and OC groups are shown in Fig. 1, accompanied by pointwise 95 % confidence intervals. Results suggest that ENDS use varies across a menstrual cycle (Fig. 1: Panel A)*,* with peaks of relative higher use co-occurring with increases in estradiol before ovulation, and with increases in estradiol and progesterone that occur before the onset of menses. In contrast, the OC group showed mostly stable ENDS use across the OC regimen (Fig. 1: Panel B).

## 4 Discussion

This proof-of-concept study highlighted the utility of an intensive longitudinal design to prospectively investigate ENDS use across a menstrual cycle and/or an OC regime. Results revealed significant variability in ENDS use in NC individuals across the menstrual cycle. The OC group did not display such variability. This pattern of results in the NC group could indicate a *positive reinforcement pathway* potentially driven by increases in estradiol, known to enhance the rewarding effects of nicotine (Wetherill et al., 2016, DeVito et al., 2014, Allen et al., 2009). The second rise in ENDS use observed during the second half of the menstrual cycle may be due to the smaller increase in estradiol that occurs in the luteal phase or influenced by negative affect. Some individuals experience affective reactivity to normal changes in hormonal levels (Eisenlohr-Moul, 2019) and be may prone to ENDS use to cope with distress (*a negative reinforcement pathway*). Indeed, several studies focused on combustible cigarettes and other substances have shown that substance use is associated with distress experienced in the late luteal phase (Sakai and Ohashi, 2013, DeBon et al., 1995, Joyce et al., 2021, Hayaki et al., 2020). In terms of the OC group, it may be that stabilized production of endogenous estradiol is associated with stable and low levels of ENDS use. This aligns with a recent study showing OC smokers had more stable use patterns and smoked fewer cigarettes than NC smokers over six weeks (Medvescek and Allen, 2023). This study has several limitations including a small sample size, lack of ethno-racial and gender diversity, and no bio-verification of hormone levels. However, although, ENDS use is difficult to measure (Soule et al., 2023), assessing “occasions” of ENDS use among NC and OC individuals is feasible. While the interpretation of results is speculative, they may serve to generate hypotheses for future studies and galvanize basic mechanistic and intervention research on the link between ovarian hormones and ENDS use. In conclusion, ENDS use among NC individuals may vary as a function of natural fluctuations in ovarian hormones while OCs appear to lower and stabilize ENDS use.

## CRediT authorship contribution statement

**Chrystal Vergara-Lopez:** Writing – original draft, Visualization, Project administration, Methodology, Investigation, Formal analysis, Data curation, Conceptualization. **Bublitz Margaret:** Writing – review & editing, Resources. **Papandonatos George:** Writing – review & editing, Visualization, Formal analysis, Conceptualization. **Stroud Laura:** Writing – review & editing, Conceptualization. **Allen Alicia:** Writing – review & editing, Methodology, Conceptualization.

## Funding

The content is solely the responsibility of the authors and does not necessarily represent the official views of the funding sources. The funding source had no other involvement other than financial support. This manuscript was supported by the 10.13039/100000026National Institute on Drug Abuse grants K08DA045935 to CVL, 5R01DA045492, 1R01DA056787, and 5R01044504 to LRS, the 10.13039/100000050National Heart, Lung, and Blood Institute R01HL157288, R01HL172869 to MHB, the 10.13039/100000057National Institute of General Medical Sciences grant P20GM139767 LRS, The 10.13039/100000066National Institute of Environmental Health Sciences 5U24ES028507 to LRS, and by a Diversity Early Career Faculty Development Award from the Department of Psychiatry and Human Behavior, The Warren Alpert Medical School of Brown University to CVL.

## Declaration of Competing Interest

The authors declare that they have no known competing financial interests or personal relationships that could have appeared to influence the work reported in this paper: Chrystal Vergara-Lopez, Margaret H. Bublitz, and Laura R. Stroud reports financial support was provided by National Institutes of Health. Authors declare that they have no known competing financial interests or personal relationships that could have appeared to influence the work reported in this paper.
